# Assessment of human exposures of cefepime-taniborbactam against cefepime-resistant Enterobacterales and *Pseudomonas aeruginosa* in a 7-day hollow fiber infection model

**DOI:** 10.1128/aac.00017-25

**Published:** 2025-07-31

**Authors:** Lindsay M. Avery, Mitchell Edwards, Fan Yi, Greg Moeck, Tsuyoshi Uehara, Daniel C. Pevear

**Affiliations:** 1 Department of Biology, Venatorx Pharmaceuticals, Inc. 540451, Malvern, Pennsylvania, USA; University of Pittsburgh School of Medicine, Pittsburgh, Pennsylvania, USA

**Keywords:** cefepime-taniborbactam, hollow-fiber infection model, carbapenem-resistant Enterobacterales, *Pseudomonas aeruginosa*

## Abstract

Taniborbactam is a novel cyclic boronate β-lactamase inhibitor that potentiates the *in vitro* activity of cefepime against Enterobacterales and *Pseudomonas aeruginosa* strains harboring serine and metallo-β-lactamases. Taniborbactam lacks intrinsic antibacterial activity. An *in vitro* hollow fiber infection model (HFIM) was used to evaluate bacterial kill and the potential for treatment-emergent resistance associated with the clinical cefepime-taniborbactam dose of 2–0.5 g every 8 h, administered as a 2 h infusion, for 7 days. Nine cefepime-resistant bacterial strains were studied among one *Escherichia coli*, five *Klebsiella pneumoniae*, and three *P. aeruginosa* that harbored a variety of cephalosporinases, extended-spectrum β-lactamases, and carbapenemases with cefepime-taniborbactam MIC values that ranged from 0.25 to 8 µg/mL. All nine strains grew rapidly when treated with cefepime alone, consistent with phenotypic resistance. Human plasma concentration-time profiles for cefepime and taniborbactam were simulated in the HFIM systems and resulted in bactericidal activity (≥3 log_10_ CFU/mL reduction) against eight of nine strains when assessed 8 h after initiation of the first dose, and against all nine strains by day 7. Treatment-emergent resistance, defined as bacterial subpopulations with ≥4 times the baseline MIC, was not detected in any cefepime-taniborbactam model from days 1 to 7. Therefore, human cefepime-taniborbactam exposures demonstrated sustained bactericidal activity and suppressed the emergence of resistance in a 7-day HFIM among serine and metallo-β-lactamase-positive Enterobacterales and *P. aeruginosa* strains. These observations support the clinical development of cefepime-taniborbactam and inform understanding of its potential role in treating serine and/or metallo-β-lactamase-positive Gram-negative bacterial infections.

## INTRODUCTION

Antibiotic resistance significantly undermines the effectiveness of common antibiotics in treating widespread bacterial infections. In 2019, bacterial antimicrobial resistance (AMR) was responsible for an estimated 1.27 million deaths worldwide and played a role in 4.95 million deaths (1). In 2024, the World Health Organization (WHO) updated and refined a Bacterial Priority Pathogens List, which ranked antibiotic-resistant bacterial pathogens in terms of the need for research and development of new antibiotics (2). Carbapenem-resistant Enterobacterales (CRE) and third-generation cephalosporin-resistant Enterobacterales remained as critical priority pathogens, similar to the preceding 2017 list. Carbapenem-resistant *Pseudomonas aeruginosa* (CRPA) was designated a high-priority pathogen.

Taniborbactam is an investigational agent that, when coupled with cefepime, remains active *in vitro* against these priority pathogens. Taniborbactam is a novel cyclic boronate β-lactamase inhibitor that potentiates the *in vitro* activity of cefepime against Enterobacterales and *Pseudomonas aeruginosa* strains harboring serine β-lactamases (SBL), types such as CTX-M, KPC, SHV, TEM, AmpC, and oxacillinase (OXA)-48, and metallo-β-lactamases (MBL), such as New Delhi metallo-β-lactamase (NDM) and Verona integron-encoded metallo-β-lactamase (VIM) (3). Taniborbactam lacks intrinsic antibacterial activity. The *in vivo* pharmacodynamics of taniborbactam in combination with cefepime have been characterized extensively in neutropenic murine thigh and kidney infection models (4, 5). These data support the use of the clinical cefepime-taniborbactam dose, 2–0.5 g every 8 h administered as a 2 h intravenous infusion, for the treatment of complicated urinary tract infections caused by β-lactamase-producing strains. In the Phase 3 CERTAIN-1 clinical trial, cefepime-taniborbactam was superior to meropenem for composite microbiologic and clinical success with a similar safety profile to meropenem in adults with complicated urinary tract infections, including acute pyelonephritis (6). Cefepime-taniborbactam is also under development for hospital-acquired bacterial pneumonia and ventilator-associated bacterial pneumonia (https://clinicaltrials.gov/study/NCT06168734).

*In vivo* data play a crucial role in understanding the pharmacodynamics of investigational antibacterial agents; however, these models are rarely used to assess the potential for the development of resistance, primarily due to the short duration of study, as translational outcomes have been linked to this 24-to-48-h timeframe (7). The hollow fiber infection model (HFIM) is a two-compartment *in vitro* infection model that can be used to simulate dynamic drug concentrations permitting the study of clinical exposure-response relationships (7). In this study, an HFIM was used to evaluate bacterial kill and the potential for treatment-emergent resistance associated with the clinical dose regimen of cefepime-taniborbactam, 2-0.5 g administered as a 2 h intravenous infusion every 8 h, against isolates of Enterobacterales and *P. aeruginosa*.

## MATERIALS AND METHODS

### Antimicrobial agents

Commercially available vials of cefepime, ceftazidime, and meropenem were used in the HFIM, while analytical powders (Sigma-Aldrich, United States Pharmacopeia) were used in susceptibility tests. Taniborbactam (Carbogen Amcis AG, Aarau, Switzerland), avibactam (prepared at Venatorx as described previously [3]), and vaborbactam (MedChemExpress, Monmouth Junction, NJ) were used throughout the study.

### Bacterial strains

Nine bacterial strains were selected to represent a variety of relevant resistance mechanisms and span a range of cefepime-taniborbactam minimum inhibitory concentration (MIC) values (0.25–8 µg/mL). This strain set was composed of clinical isolates including one *Escherichia coli*, five *Klebsiella pneumoniae,* and three *P. aeruginosa* that were originally sourced from the Centers for Disease Control and Prevention and U.S. Food and Drug Administration Antimicrobial Resistance (AR) Isolate Bank (Atlanta, GA) https://www.fda.gov/medical-devices/in-vitro-diagnostics/cdc-fda-antibiotic-resistance-isolate-bank, American Type Culture Collection (ATCC; Manassas, VA), or the International Health Management Associates (Schaumburg, IL). Whole-genome sequencing (WGS) was performed for six strains to confirm strain identity and the presence of genes/mutations that are known to be involved in β‑lactam resistance. The publicly available genomes of the remaining three strains (ATCC BAA‑1705, AR 0054, and AR 0357) were also analyzed. Full description of the methods for WGS analysis is included in Supplementary Appendix A.

### Antibiotic susceptibility testing

MIC modal values were determined by broth microdilution according to CLSI (M07) methods (8); recommended quality control (QC) strains were included for each agent. Cefepime and cefepime-taniborbactam MICs were determined by broth microdilution using the inoculum prepared for the HFIM assessment of cefepime-taniborbactam. The HFIM inoculum was diluted to the concentration for MIC testing by broth microdilution (i.e., 10^5^ CFU/mL). This step confirmed that the intended strain was prepared and inoculated into the intended bioreactor (HFIM cartridge) and that the known β-lactamase resistance mechanisms were active in the HFIM system at the time of the study.

### Hollow fiber infection model (HFIM)

The HFIM was constructed as described previously (7). In the HFIM, bacteria were exposed over time to clinically relevant, fluctuating drug concentrations achieved by repeated dosing and constant elimination. Twenty milliliters of a log phase bacterial suspension was used to inoculate the extracapillary space of the hollow fiber cartridges (C2011, FiberCell Systems, Frederick, MD). Programmable syringe pumps (New Era Pump Systems, Inc., NE-1600) infused all drugs (every 8 h) into the central reservoir to recapitulate human plasma exposures. The contents of the hollow fiber cartridge were rapidly equilibrated with those of the central reservoir via a Duet pump (FiberCell Systems, Frederick, MD) set at a maximum rate of approximately 130 mL/min. The drug concentration-time profile in the entire two-compartment system was governed by the infusion of sterile, non-drug-supplemented growth media into the central reservoir, while the volume was held constant by the removal of drug-containing broth at an equal rate. Studies were conducted over a 7-day period. Cefepime-taniborbactam (2–0.5 g every 8 h, 2 h infusion) was the primary regimen evaluated. Cefepime monotherapy (2 g every 8 h, 2 h infusion) served as growth controls in all experiments. Except for growth controls, sampling ceased when the models reached maximum visual turbidity consistent with regrowth to approximately 10 log_10_ CFU/mL.

Qualification experiments of the HFIM with commercially available β-lactam/β-lactamase inhibitor combinations were performed. A total of three study strains and two commercially available β-lactam/β-lactamase inhibitors, ceftazidime-avibactam and meropenem-vaborbactam, were used. Ceftazidime-avibactam 2–0.5 g every 8 h (2 h infusion) was evaluated against *K. pneumoniae* ATCC BAA-1705 (KPC-2 positive; ceftazidime-avibactam MIC, 1 µg/mL), and meropenem-vaborbactam 2–2g every 8 h (3 h infusion) was assessed against *K. pneumoniae* 752285 (OXA-48-positive; meropenem-vaborbactam MIC, 2 µg/mL). Against an MBL-producing strain (*P. aeruginosa* AR 0054 expressing VIM-4; ceftazidime-avibactam MIC, 128 µg/mL), EDTA was added to HFIM growth media (in both the central reservoir and extracapillary space) at a fixed concentration (20 µg/mL) previously demonstrated to restore susceptibility to ceftazidime-avibactam in a broth microdilution assay (i.e., ceftazidime-avibactam MIC ≤8 µg/mL [9]). EDTA sequesters zinc contained in cation-adjusted Mueller-Hinton broth (CAMHB), which is required by the VIM-4 MBL to hydrolyze β-lactam substrates, such as ceftazidime.

### Bacterial preparation

Models were inoculated with 20 mL of a log phase bacterial suspension (>10^7^ CFU/mL, >10^8^ total CFU) up to 1 h prior to hour 0. The suspension was prepared by inoculating 10 mL of sterile CAMHB with one isolated colony of the test strain. Following overnight incubation at 37°C, a 1 mL subculture was added to 10 mL of sterile CAMHB, re-incubated for approximately 1 h, and the target density was adjusted according to measurement of an OD at 600 nm. Once inoculated, the bacterial contents of the hollow fiber culture in the extracapillary space were mixed using sterile syringes. The cartridge contents were also mixed immediately prior to sampling from this infection compartment.

### CFU determination

The activities of cefepime monotherapy (i.e., cefepime-alone), cefepime-taniborbactam, ceftazidime-avibactam, and meropenem-vaborbactam human exposures were monitored by quantitative culture. Bacterial densities at sampling time points (0, 6–8, 24, 48, 72, 96, 120, 144, and 168 h) were determined using an automatic dilutor and spiral plater (easySpiral Dilute, Interscience, Woburn, MA, USA) from samples collected from the extracapillary space in approximately 1 mL volumes. Samples were also plated manually onto cation-adjusted Mueller-Hinton agar (CAMHA) supplemented with four times the MIC (4 × MIC) of the β-lactam/β-lactamase inhibitor to monitor for the emergence of subpopulations with elevated MICs from baseline in the HFIM experiments. This method was previously used to detect colonies with elevated cefepime-taniborbactam MIC values (3). The β-lactamase inhibitor concentration was the same in all plates to reflect the fixed concentration in the reference broth microdilution method for the β-lactam/β-lactamase inhibitor combination (i.e., 4 µg/mL avibactam or taniborbactam, 8 µg/mL vaborbactam). The lower limit of detection was 1.7 log_10_ CFU/mL (i.e., 5 CFU in 100 µL) except at 168 h, for which the lower limit was 0.7 log_10_ CFU/mL (i.e., 5 CFU in 1 mL; 100 µL on each of 10 plates).

### Dosage regimens and pharmacokinetic (PK) evaluation

Target-free (unbound to plasma proteins) drug PK profiles of cefepime-taniborbactam (2–0.5 g every 8 h, 2 h infusion) were based on population parameters derived from healthy volunteers (10). Free ceftazidime-avibactam (2–0.5 g every 8 h, 2 h infusion) and meropenem-vaborbactam (2–2g every 8 h, 3 h infusion) plasma profiles were informed by prescribing information and published data, respectively (11, 12). Doses administered to the HFIM were calculated to achieve their respective target PK profile according to a constant media diluent rate. This HFIM clearance rate was maintained across all experiments at approximately 0.08 L/h (1.7 h half-life) to optimize recapitulation of cefepime and taniborbactam concentration-time profiles as the pharmacokinetics of cefepime and taniborbactam are similar in humans (10). A comparison of human PK profiles to those targeted in the HFIM is found in Table 1.

**TABLE 1:** Comparison of human pharmacokinetic profiles to those targeted in the hollow fiber infection model (HFIM)^*d*^^,^^*e*^

Clinical dose simulation		Free time (%) above MIC of:							*f*AUC_0-24_ (µg/mL*h)	*f*C_max,0-24_ (µg/mL)
		0.5	1	2	4	8	16	32		
Cefepime 2 g q8h, 2 h infusion	HFIM	100	100	100	100	85	61	34	620	62
	Human^*a*^	100	100	100	100	85	59	35	688	78
Taniborbactam 0.5 g q8h, 2 h infusion	HFIM	100	100	94	71	46	15	0	208	21
	Human^*a*^	100	100	92	70	46	17	0	208	21
Ceftazidime 2 g q8h, 2 h infusion	HFIM	100	100	100	100	84	62	38	676	68
	Human^*b*^	100	100	100	100	93	70	44	771	76
Avibactam 0.5 g q8h, 2 h infusion	HFIM	100	91	71	48	20	0	0	110	11
	Human^*b*^	92	77	61	44	22	0	0	106	13
Meropenem 2 g q8h, 3 h infusion	HFIM	100	100	100	100	80	58	31	550	52
	Human^*c*^	100	100	100	97	77	57	32	551	51
Vaborbactam 2 g q8h, 3 h infusion	HFIM	100	100	100	97	77	55	25	494	46
	Human^*c*^	100	100	100	100	83	58	23	494	42

^*a*^ Reference (10).

^*b*^ Reference (11).

^*c*^ Reference (12).

^*d*^ fAUC_0-24_, free drug (unbound to plasma proteins) area under the concentration-time curve from time 0 to 24 h; *f*C_max_,_0-24_, free drug (unbound to plasma proteins) maximum concentration from time 0 to 24 h; HFIM, hollow fiber infection model; h, hour; MIC, miminum inhibitory concentration; q, every.

^*e*^ Concentrations represent free drug (unbound to plasma proteins): Protein binding values used were ceftazidime (10%), avibactam (7%), meropenem (2%), vaborbactam (33%), cefepime (22.4%), and taniborbactam (0%).

PK samples were collected from the central circulation to confirm β-lactam and β-lactamase inhibitor concentrations. Studies of other β-lactam/β-lactamase inhibitor combinations have demonstrated variable levels of β-lactam degradation by β-lactamase-producing bacteria in the HFIM that may not align with the β-lactam dose administered (13). Therefore, β-lactam target concentrations over time were confirmed during preliminary cartridge compatibility studies in non-infected media. Confirmatory pharmacokinetic (PK) analyses were performed only for the β-lactamase inhibitors during the conduct of the HFIM studies. Acceptable pharmacokinetic exposure of taniborbactam was defined by (i) first-dose peaks (i.e., immediately after the end of the first infusion) that were less than 120% the target clinical value and (ii) all trough values measured below the estimated 90th percentile clinical value at hour 8 (3.0 μg/mL) according to phase 1 data (10). Concentrations of avibactam and vaborbactam in the central compartments of HFIMs were assessed over time to confirm each profile recapitulated human PK.

### Bioanalytical assay

Analytes (cefepime, ceftazidime, meropenem, taniborbactam, avibactam, and vaborbactam) were quantified by fit-for-purpose, qualified bioanalytical methods. Full description of the bioanalytical methods for each analyte is provided in Supplementary Appendix B. In brief, samples collected from cartridge compatibility studies were analyzed by Syneos Health (Princeton, New Jersey). Analytes were quantified over a range of 0.1–100 µg/mL using a high-performance liquid chromatography tandem mass spectrometry (LC-MS/MS) method. Confirmatory PK samples in the HFIM assessments for each strain were analyzed in-house with a qualified method. Analytes were quantified over a range of 0.05 to 50 µg/mL (0.1 to 50 µg/mL for avibactam) using an ultraperformance LC-MS/MS method (Waters). All developed assays were qualified with accuracy and precision tested over the method range. Samples were stored at −80°C prior to assay; meropenem and vaborbactam samples were stabilized in an equal volume of 1 M MOPS buffer (pH 7) prior to freezing.

## RESULTS

### Antimicrobial susceptibility testing and molecular characterization

Modal MIC values (determined from three to four replicates) of cefepime (range, 16–128 µg/mL), cefepime-taniborbactam (0.25–8 µg/mL), ceftazidime-avibactam (0.25–>128 µg/mL), and meropenem-vaborbactam (0.016–>128 µg/mL) against the nine strains studied are provided in Table 2. The results of the whole-genome sequencing and genomic analyses for these strains are also provided in Table 2. Four strains contained MBLs (two NDM-1, one VIM-1, and one VIM-4), six strains harbored extended-spectrum β-lactamases (ESBLs; two CTX-M-15, one SHV-12, one SHV-30, one VEB-1, one PER-1), and three strains contained serine carbapenemases (two KPC-2, one OXA-48). Four strains co-harbored ESBL and carbapenemase. Alterations in penicillin-binding protein 3 (PBP3) were noted in two strains (Table 2).

**TABLE 2:** Characterization of strains assessed in the hollow fiber infection model^*j*^

Bacterial isolate	MBL	Serine β- lactamases	Outer membrane porin variants	PBP3	Broth microdilution MIC (µg/mL)					
					FEP	FTB	CAZ	CZA	MEM	MVB
*E. coli* AR 0055	NDM-1	EC-690, CMY-6, OXA-1	OmpC: Intact OmpF: Intact	A233T, I332V	>128	2	>128	>128	64	64
*K. pneumoniae* 752285	Not found	CTX-M-15, SHV-11, TEM-1, OXA-9, OXA-48	OmpK35: Intact OmpK36: Lesion^*a*^	WT	>128	0.25	64	0.5	4	2
*K. pneumoniae* AR 0126	Not found	KPC-2, SHV-1, SHV-30, TEM-1, OXA-1	OmpK35: Intact OmpK36: Lesion^*b*^	WT	16	1	8	0.25	8	0.03
*K. pneumoniae* AR 0135	VIM-1	SHV-12, TEM-1, OXA-9	OmpK35: Lesion^*c*^ OmpK36: Intact	WT	128	1	>128	>128	8	8
*K. pneumoniae* AR 0145	NDM-1	CTX-M-15, SHV-11, TEM-1, OXA-1, OXA-9	OmpK35: Lesion^*d*^ OmpK36: Intact	WT	128	0.25	>128	>128	64	64
*K. pneumoniae* ATCC BAA-1705^*e*^	Not found	KPC-2, SHV-11, truncated OXA-9	OmpK35: Lesion^*f*^ OmpK36: Intact	WT	32	0.25	128	1	32	0.016
*P. aeruginosa* 2235344	Not found	PDC-1, OXA-847, PER-1, OXA-2	OprD: Lesion^*g*^	WT	32	2	>128	8	16	16
*P. aeruginosa* AR 0054^*e*^	VIM-4	PDC-3, OXA-396	OprD: Lesion^*h*^	F533L	128	8	128	128	>128	>128
*P. aeruginosa* AR 0357^*e*^	Not found	PDC-35, OXA-488, VEB-1, OXA-10	OprD: Lesion^*i*^	WT	128	8	>128	8	8	8

^*a*^ Frameshift by a nonsense mutation (stop codon) at codon 311.

^*b*^ Frameshift by one base pair (bp) deletion at codon 231 of OmpK36 porin (NCBI ID: WP_009484383.1).

^*c*^ Frameshift by 1 bp deletion at codon 61.

^*d*^ Frameshift by 7 bp insertion at codon 56.

^*e*^ Genome sequencing was not performed as publicly available genomes were analyzed.

^*f*^ Frameshift by 1 bp insertion at codon 40.

^*g*^ Frameshift by 1 bp deletion at codon 318.

^*h*^ Frameshift by a nonsense mutation (stop codon) at codon 415.

^*i*^ Frameshift by 5 bp insertion at codon 404.

^*j*^ AR, denotes a strain sourced from the Centers for Disease Control and Prevention and Food and Drug Administration Antimicrobial Resistance (AR) isolate bank; ATCC, American Type Culture Collection; CAZ, ceftazidime; CZA, ceftazidime-avibactam; EC, *Escherichia coli*; FEP, cefepime; FTB, cefepime-taniborbactam; MBL, metallo-β-lactamase; MEM, meropenem; MVB, meropenem-vaborbactam; MIC, minimum inhibitory concentration; NDM, New Delhi metallo-β-lactamase; OmpC, outer membrane porin C; OprD, outer membrane porin D; OmpF, outer membrane porin F; OmpK, outer membrane porin K; PBP3, penicillin-binding protein 3; VIM, Verona integron-encoded metallo- β-lactamase; WT, wild type.

### HFIM qualification studies

**Fig 1:**
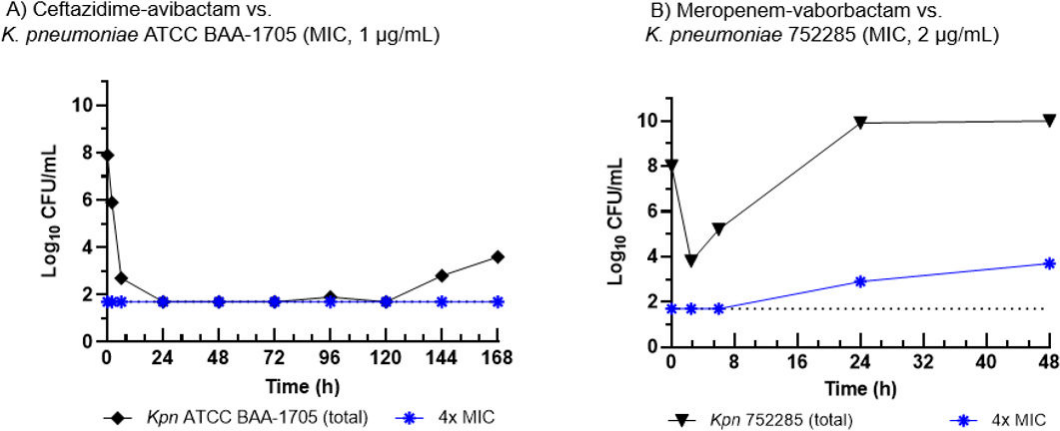
Hollow fiber infection model qualification experiments.Legend: HFIM experiments evaluating the activity of either ceftazidime-avibactam (A) or meropenem-vaborbactam (B) against each individual strain (black filled circles). Resistant subpopulations that grew on 4 × MIC CAMHA following treatment (blue star) are depicted. Abbreviations: ATCC, American Type Culture Collection; CFU, colony forming unit; *Kpn*, *Klebsiella pneumoniae*; MIC, minimum inhibitory concentration

The HFIM was qualified by assessing the activity of two marketed β-lactam/β-lactamase inhibitor combinations against two SBL-producing strains and one MBL-producing strain. When the KPC-2-producing reference strain *K. pneumoniae* ATCC BAA-1705 (ceftazidime-avibactam MIC, 1 µg/mL) was challenged with simulated human exposures of ceftazidime-avibactam (Fig. 1A), bactericidal activity was achieved early during the first dosing interval and was maintained throughout 7 days (168 h). Despite a trend toward regrowth beginning at hour 144, a subpopulation with an elevated ceftazidime-avibactam MIC was not detected, and the overall pharmacodynamic effect was consistent with *in vitro* susceptibility testing results. When OXA-48-producing *K. pneumoniae* 752285 (meropenem-vaborbactam MIC, 2 µg/mL) was challenged with simulated human exposures of meropenem-vaborbactam, bioburden reduction of 4.2 log_10_ CFU/mL was observed at hour 2.5 (Fig. 1B), and no colonies arose on agar supplemented with 4 × MIC concentrations of meropenem-vaborbactam at baseline through hour 6. However, significant regrowth became apparent at the 6 h time point, and maximum culture density was achieved by hour 24. Once the culture reached densities of 9.9 log_10_ CFU/mL and 10.0 log_10_ CFU/mL at hours 24 and 48, respectively (as quantified on non-selective CAMHA), the same samples exhibited 2.9 log_10_ CFU/mL at hour 24 and 3.7 log_10_ CFU/mL at hour 48 on agar supplemented with 4 × MIC concentrations of meropenem-vaborbactam, indicating growth of a resistant subpopulation. Qualification was also performed with an MBL-positive strain, *P. aeruginosa* AR 0054 (VIM-4-positive), treated with ceftazidime-avibactam in CAMHB supplemented with or without EDTA (i.e., zinc-chelator) to examine whether the activity of MBL impacts the efficacy of ceftazidime-avibactam. A lower starting inoculum of 10^6^ CFU/mL (i.e., >10^7^ total CFU) was qualified for use with this strain. Full details regarding the qualification of this strain are provided in Supplementary Appendix C.

### HFIM studies with cefepime-taniborbactam

**Fig 2:**
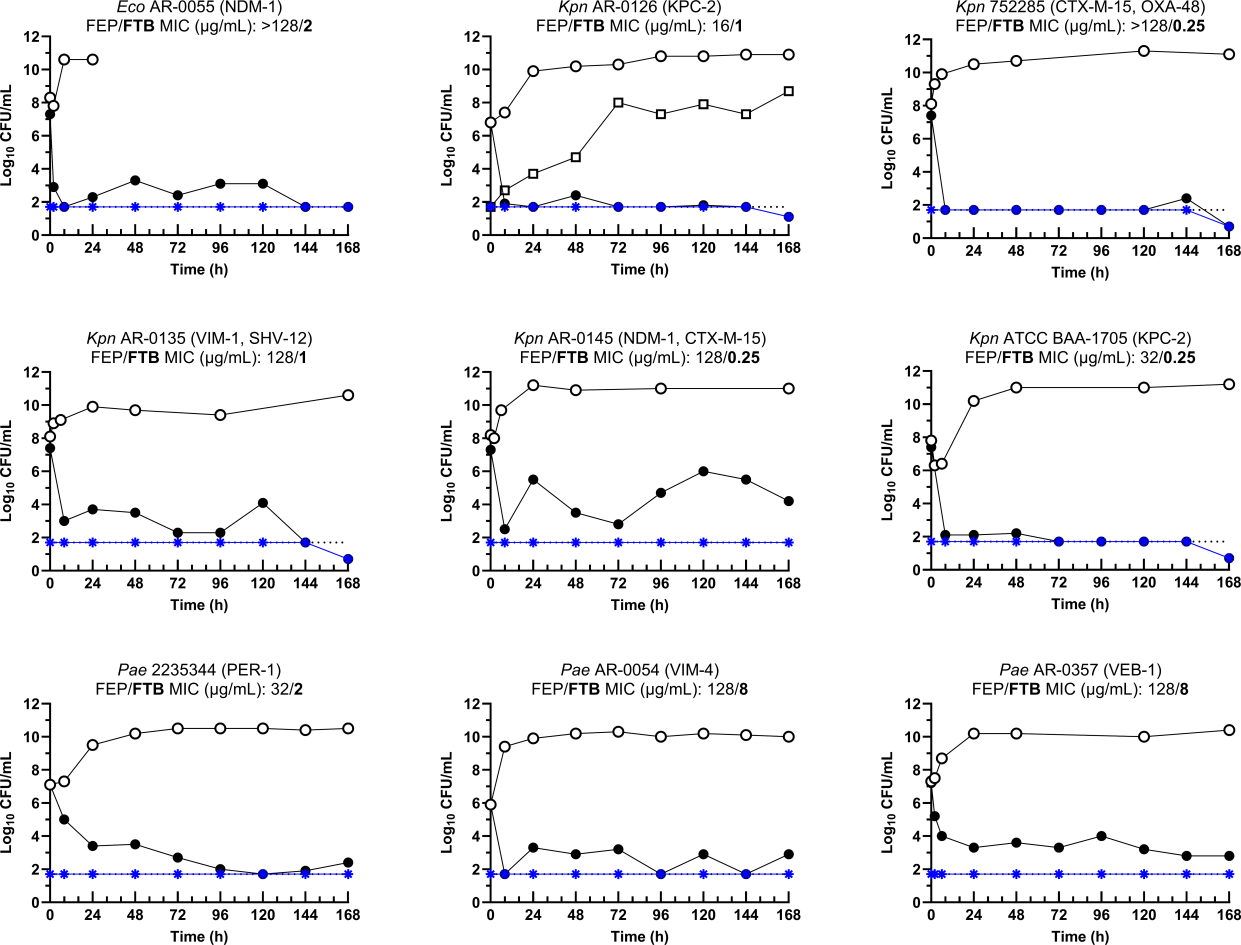
Activities of cefepime and cefepime-taniborbactam against Enterobacterales (*N* = 6) and *P. aeruginosa* (*N* = 3) assessed in the hollow fiber infection model. Legend*:* The CFU data obtained in HFIM experiments evaluating the activity of cefepime alone (black, open circle) and cefepime-taniborbactam (black, filled circle) human exposure are plotted for each individual strain. Subpopulations that grew on 4 × cefepime-taniborbactam MIC supplemented CAMHA following treatment with cefepime (black, open square) or cefepime-taniborbactam (blue star) are depicted. Notably, black open squares are not shown (except for strain *Kpn* AR-0126) for simplicity, as they were at the lower limit of detection. Abbreviations: AR, denotes a strain sourced from the Centers for Disease Control and Prevention and Food and Drug Administration Antimicrobial Resistance (AR) Isolate Bank; ATCC, American Type Culture Collection; CFU, colony forming unit; *Eco*, *E. coli*; FEP, cefepime; FTB, cefepime-taniborbactam; HFIM, hollow fiber infection model; *Kpn*, *Klebsiella pneumoniae*; MIC, minimum inhibitory concentration; NDM, New Delhi metallo-β-lactamase; *Pae*, *Pseudomonas aeruginosa*; VIM, Verona integron-encoded metallo-β-lactamase

Starting inocula ranged from 6.8 to 7.4 log_10_ CFU/mL for eight strains and 5.9 log_10_ CFU/mL for the remaining *P. aeruginosa* AR 0054. Bacterial burden data are summarized in Table 3. All strains grew greater than 1 log_10_ CFU/mL from the initial inoculum by hour 24 when treated with simulated human exposures of cefepime monotherapy, and bioburdens were maintained near 10 log_10_ CFU/mL for the entirety of the experiments (Fig. 2). The addition of simulated human taniborbactam exposures to the simulated human cefepime dose resulted in bactericidal activity (≥3 log_10_ CFU/mL reduction) against eight strains when assessed 8 h after initiation of the first dose, and against all nine strains at 168 h (Fig. 2; Table 3). In the HFIM with *K. pneumoniae* AR 0126, from the cefepime-alone treatment arm, 8.7 log_10_ CFU/mL was detected on the 4 × MIC cefepime-taniborbactam plates at the final 168 h time point, while 10.9 log_10_ CFU/mL was detected on non-supplemented plates (Fig. 2). The subpopulation appeared to grow slowly, and this definitive pattern was not detected for any of the other strains. On the contrary, treatment-emergent resistance was not detected in any cefepime-taniborbactam treated model from days 1 to 7 as no colonies arose on 4 × MIC plates.

**TABLE 3:** Summary of *in vitro* activities of simulated human exposures of cefepime-taniborbactam (2–0.5 g every 8 h administered as a 2 h intravenous infusion) against bacterial strains (*N* = 9) assessed in the hollow fiber infection model^*d*^

Strain identifier	MIC (µg/mL)		Change in bacterial burden (log_10_ CFU/mL) from 0 h to timepoint			Recovery of CFU with increased MIC*^a^*
	FTB^*b*^	FEP	8 h	24 h	168 h	
*E. coli* AR 0055	2	>128	−5.6	−5.0	−5.6	No
*K. pneumoniae* 752285	0.25	>128	−5.7	−5.7	−6.7	No
*K. pneumoniae* AR 0126	1	16	−4.9	−5.3	−5.7	No
*K. pneumoniae* AR 0135	1	128	−4.4	−3.7	−6.7	No
*K. pneumoniae* AR 0145	0.25	128	−4.8	−1.8	−3.1	No
*K. pneumoniae* ATCC BAA-1705	0.25	32	−5.3	−5.3	−6.7	No
*P. aeruginosa* 2235344	2	32	−2.1	−3.7	−4.7	No
*P. aeruginosa* AR 0054	8	128	−4.2	−2.6	−3.0	No
*P. aeruginosa* AR 0357	8	128	−3.2^*c*^	−3.9	−4.4	No

^*a*^ Emergence of resistance was determined on CAMHA supplemented with 4 × MIC of cefepime-taniborbactam.

^*b*^ Minimum inhibitory concentration of cefepime by broth microdilution in the presence of a fixed concentration of 4 µg/mL taniborbactam.

^*c*^ Sample collected early at hour 6, 2 h prior to the end of the first 8 h dosing interval.

^*d*^ AR, denotes a strain sourced from the Centers for Disease Control and Prevention and Food and Drug Administration Antimicrobial Resistance (AR) Isolate Bank; ATCC, American Type Culture Collection; CFU, colony forming unit; FEP, cefepime; FTB, cefepime-taniborbactam; h, hour; HFIM, hollow fiber infection model; MIC, minimum inhibitory concentration.

### PK evaluation

β-Lactam target concentrations over time were confirmed during preliminary cartridge compatibility studies in non-infected media. These studies were conducted for all analytes to confirm that targeted concentrations were achieved in the central reservoir and that equilibration with the extracapillary space of the cartridge was achieved. The concentration-time profile for cefepime is displayed in Fig. S2. β-Lactam PK profiles achieved free drug time above the MIC (*f*T >MIC) values of 100% up to 2 µg/mL; *f*T >MIC at 4, 8, and 16, respectively, were 100%, 81%, and 60% for cefepime; 100%, 84%, and 63% for ceftazidime; and 93%, 71%, and 45% for meropenem; demonstrating comparable profiles to human values (Table 1).

**Fig 3:**
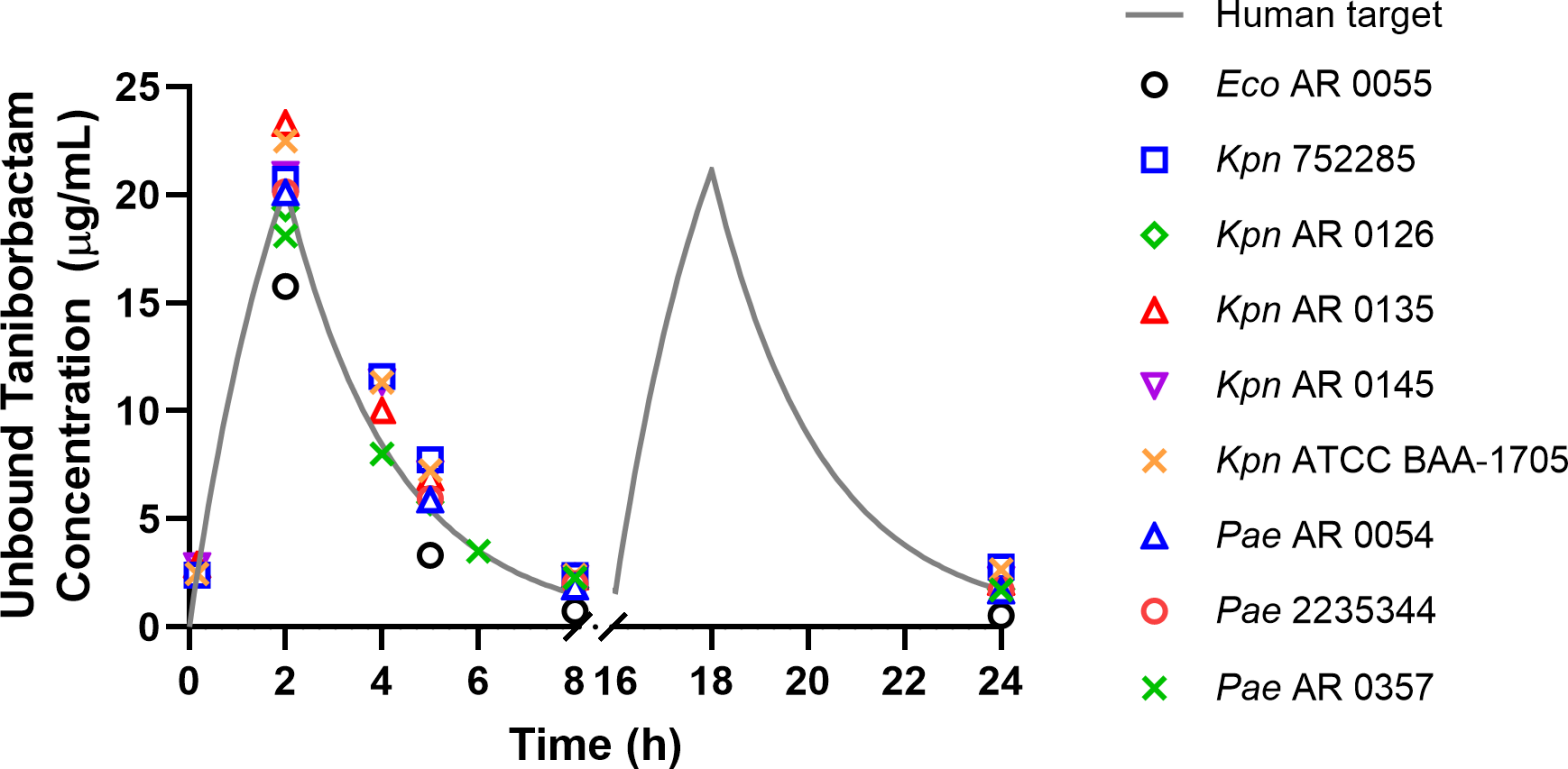
Taniborbactam clinical target concentration-time profile and measured concentrations in cefepime-taniborbactam HFIM assessments. Abbreviations: AR, denotes a strain sourced from the Centers for Disease Control and Prevention and Food and Drug Administration Antimicrobial Resistance (AR) Isolate Bank; ATCC, American Type Culture Collection; *Eco*, *E. coli; Kpn*, *Klebsiella pneumoniae; Pae*, *Pseudomonas aeruginosa*

The clinical taniborbactam concentration-time profile was achieved in the HFIM against all strains evaluated. The measured concentrations in the HFIM studies for Enterobacterales and *P. aeruginosa* strains were overlaid on the clinical target exposure profile in Fig. 3. All peak values ranged between 77.41% and 114.44% of the target value (10). All trough values were less than 3.0 µg/mL and ranged between 0.53 and 2.79 µg/mL. For avibactam and vaborbactam, the intended pharmacokinetic exposures were similar to the targeted human PK as displayed in Fig. S3.

## DISCUSSION

CRE, third-generation cephalosporin-resistant Enterobacterales, and CRPA pose a high estimated burden among multidrug-resistant (MDR) Gram-negative bacteria due to their widespread prevalence and resistance (2). Cefepime-taniborbactam is an investigational agent with activity against these MDR organisms, and thorough investigation to clearly define the utility of this newer agent is clinically important. The most preferred method of investigating the activity of a novel antibacterial agent is through the conduct of a randomized, controlled clinical trial. However, it can be extremely challenging to find and recruit enough patients with infections caused by MDR pathogens, the exact patients of most interest to the scientific community for evaluating newer agents (7). Thus, critical information regarding the activity of new agents against MDR pathogens is often gleaned through rigorous assessment in established *in vitro* models. One such model, the HFIM, is a two-compartment *in vitro* infection model that can be used to simulate dynamic drug concentrations permitting the study of clinical exposure-response relationships (7). This model has wide applications in the field of biomedicine (14), and it is considered the preferred *in vitro* infection model for studies of antibacterial pharmacokinetic/pharmacodynamic indices (7). A major benefit over the one-compartment dynamic *in vitro* infection model (i.e., chemostat) is the HFIM’s ability to accommodate prolonged treatment courses for assessments of the potential for treatment-emergent resistance. In addition, the HFIM avoids a pitfall of the chemostat, in which the waste contains bacterial cells which could lead to underestimation of the drug exposure needed for bacterial kill and resistance prevention. A 7-day/168 h treatment duration was selected for the present evaluation of cefepime-taniborbactam, as it represents a typical treatment duration for serious infections caused by the pathogens studied. However, a wide range of durations is reported in recent investigations, from as few as 32 h (12) to weeks (15).

In the current study, simulated human exposures of taniborbactam with cefepime resulted in bactericidal activity (≥3 log_10_ CFU/mL reduction) against all nine strains by day 7. Additionally, no treatment-emergent resistance was detected in any of the cefepime-taniborbactam-treated models from days 1 to 7. Moreover, in the assessment of *K. pneumoniae* AR 0126, a subpopulation with an elevated cefepime-taniborbactam MIC (from 1 to 8 µg/mL) was detected in the outgrowth from the cefepime-alone treatment arm. This pattern was not detected in any of the other eight strains evaluated, suggesting that the cefepime-taniborbactam treatment arm was able to suppress both emergence of resistance and outgrowth of pre-existing mutants in the inoculum. These findings are particularly noteworthy given that the strains evaluated were chosen to represent clinically relevant bacterial pathogens (i.e., CRE, third-generation cephalosporin-resistant Enterobacterales, and CRPA) exhibiting a wide range of potential resistance mechanisms that affect cefepime-taniborbactam activity, including various SBLs, MBLs, porin mutations, and combinations of multiple resistance mechanisms. Moreover, the robustness of these results is underscored by the challenging conditions of the HFIM, which involved a starting inoculum (>7–8 log_10_ CFU total) significantly higher than the 10^5^ CFU/mL (approximately 6 log_10_ CFU total) typically used in routine *in vitro* susceptibility assessments. The absence of treatment-emergent resistance with cefepime-taniborbactam over the 7-day/168 h experiments is especially striking compared to the performance of meropenem-vaborbactam against *K. pneumoniae* 752285. Despite a susceptible MIC of 2 µg/mL by broth microdilution (0.1 mL), simulated human meropenem-vaborbactam exposures failed to prevent growth in the hollow fiber extracapillary space (>10 mL), likely due to expression of OXA-48, which can inactivate meropenem but is not inhibited by vaborbactam. Similar results were reported also in a neutropenic murine thigh infection model (16).

The activity of cefepime-taniborbactam against *K. pneumoniae* AR-0145 is also worth mentioning. As displayed in Fig. 2, a rapid reduction in bacterial burden was observed following administration of cefepime-taniborbactam, followed by regrowth to between 4 and 6 log_10_ CFU/mL from hours 96 to 168. A mechanistic rationale to explain this regrowth could not be provided as it was not investigated further. However, no growth was observed on CAMHA supplemented with 4 × MIC cefepime-taniborbactam throughout the experiment, indicating that the regrowth observed was not mediated by the emergence of a cefepime-taniborbactam resistant subpopulation. This observation highlights the value of performing HFIM studies on several strains that represent a clinically relevant bacterial population, as slight variation in the pharmacodynamic profile is often observed even for isolates with MICs within a susceptible range. Future work could assess mechanisms influencing the various pharmacodynamic effects that are not explained by the MIC.

There are some limitations to this study. First, all HFIM experiments were performed in singlicate, similar to other HFIM studies in the literature (17, 18). We chose to investigate the activity of cefepime-taniborbactam against a diverse set of cefepime-resistant strains rather than perform multiple replicates with fewer strains. Second, the targeted drug concentration-time profile was based on healthy volunteer PK and not PK from infected patients. This is common for HFIM studies in the literature, especially at the earlier stages of drug development when PK in critically ill patients is lacking (12, 19). Third, while β-lactam target concentrations over time were confirmed during preliminary cartridge compatibility studies in non-infected media, they were not confirmed during the conduct of the HFIM studies; only β-lactamase inhibitors were measured. Monitoring for β-lactam degradation may be useful to elucidate relationships between taniborbactam concentration and resistance prevention thresholds; however, this was beyond the scope of the current study. Another limitation common to all HFIM and chemostat models is the lack of immune cells and conditions of infection sites, which neglects the effect of the immune system and other human factors in combating infection. With *in vitro* infection models, bacterial cultures reside in environments enriched for persistence (e.g., regular replenishment of artificial growth media, means of gas and waste exchange). However, this could also be considered an advantage to allow investigation of the effects of the antibiotics alone, without interference by immune cells.

### Conclusion

Cefepime-taniborbactam suppressed the emergence of resistance against nine ESBL and/or carbapenemase-producing, cefepime-resistant Enterobacterales and *P. aeruginosa* strains in a qualified HFIM following 7 days of treatment with simulated human exposures equivalent to the clinical dose (cefepime-taniborbactam: 2–0.5 g every 8 h, 2 h infusion). Regrowth to the level of the initial inoculum (>7–8 log_10_ CFU) was prevented for the entire treatment duration, with ≥3 log_10_ CFU/mL reductions (i.e., bactericidal response) observed at day 7 for all nine strains. These observations support further clinical development of cefepime-taniborbactam and inform understanding of its potential role in the treatment of infections caused by cefepime- and carbapenem-resistant strains of Enterobacterales and *P. aeruginosa*, including strains producing serine- and metallo-β-lactamases.

## Data Availability

Raw sequence reads for the genome-sequenced strains listed in Table 2 were deposited in GenBank under BioProject accession number PRJNA1242862.

## SUPPLEMENTAL MATERIAL

Supplemental material aac.00017-25-s0001.docx Table S1; Fig. S1 to S3.

ASM does not own the copyrights to Supplemental Material that may be linked to, or accessed through, an article. The authors have granted ASM a non-exclusive, world-wide license to publish the Supplemental Material files. Please contact the corresponding author directly for reuse.
