# Supplementary material for: Assessment of human exposures of cefepime-taniborbactam against cefepime-resistant Enterobacterales and Pseudomonas aeruginosa in a 7-day hollow fiber infection model
Source: Antimicrob Agents Chemother. 2025 Jul 31;69(9):e00017-25. doi: 10.1128/aac.00017-25 (PMC12406669; doi:10.1128/aac.00017-25)
Supplement: Supplemental material — Table S1; Fig. S1 to S3. [file aac.00017-25-s0001.docx]

**Supplementary Appendix A: Whole Genome Sequencing Analysis**

For the WGS of the six strains, cells were prepared from untreated (baseline) cultures. DNA extraction, library preparation, Illumina HiSeq and generation of raw FASTQ sequence data (150-bp paired-end reads) were performed by GeneWiz (South Plainfield, NJ). The analyses of FASTQ files were performed using Geneious Prime version 2022.1.1 (Biomatters Inc., San Diego, CA). The reads were trimmed with BBDUK Adapter/Quality Trimming Version 38.84 (Brian Bushnell), yielding 6 to 12 million reads per genome. For the four strains whose reference genomes were available at the NCBI database (strains AR 0055, AR 0126, AR 0135 and AR 0145), trimmed reads were aligned to the corresponding reference genome and genetic variation was investigated. For *K. pneumoniae* 752285 and *P. aeruginosa* 2235344, de novo assembly was performed with the Geneious Assembler. β-lactamases in each genome were annotated using a search set of 84 representative β-lactamases and cutoff of 40% identity. This search set successfully identifies all ~2,000 β-lactamases included in ResFinder. If an annotated β-lactamase gene was incomplete or split over two contigs or had a premature stop codon, 100% of the trimmed reads were mapped to the standard sequence and then analyzed further to verify the presence of an intact gene, truncated gene, or multiple alleles. The *ftsI* gene encoding PBP3 and the genes encoding major porins (OmpC/OmpK36, OmpF/OmpK35, and OprD) were annotated using the reference genes/proteins listed in **Table S1.** The publicly available genomes of the remaining three strains (ATCC BAA-1705, AR 0054, and AR 0357) were also analyzed as described above.

**Table S1: Reference Genes Utilized in the Whole Genome Sequencing Analysis**

| **Organism** | **Strain** | **Gene Name (protein name)** | **Nucelotide Accession** | **Locus Tag/**  **Protein-Coding DNA Sequence** | **Protein Accession** |
| --- | --- | --- | --- | --- | --- |
| *E. coli* | MG1655 | *ompC* | NC_000913.3 | b2215 | NP_416719.1 |
| *E. coli* | MG1655 | *ompF* | NC_000913.3 | b0929 | NP_415449.1 |
| *E. coli* | MG1655 | *ftsI* (PBP3) | NC_000913.3 | b0084 | NP_414626.1 |
| *K. pneumoniae* | ATCC 13883 | *ompK36* | NZ_KN046818 | DR88_RS05545 | WP_004149145.1 |
| *K. pneumoniae* | ATCC 13883 | *ompK35* | NZ_KN046818 | DR88_RS17700 | WP_004195943.1 |
| *K. pneumoniae* | ATCC 13883 | *ftsI* (PBP3) | NZ_KN046818 | DR88_RS22130 | WP_002888559.1 |
| *P. aeruginosa* | PAO1 | *oprD* | NC_002516.2 | PA0958 | NP_249649.1 |
| *P. aeruginosa* | PAO1 | *ftsI* (PBP3) | NC_002516.2 | PA4418 | NP_253108.1 |

**Supplementary Appendix B: Bioanalytical Analysis**

Analytes (cefepime, ceftazidime, meropenem, taniborbactam, avibactam, and vaborbactam) were quantitated by fit-for-purpose, qualified bioanalytical methods. Samples collected from cartridge compatibility studies were analyzed by Syneos Health (Princeton, New Jersey). All confirmatory pharmacokinetic samples in the HFIM assessments for each strain were analyzed at Venatorx with an internal method, except for *Eco* AR 0055 that was processed by Syneos Health. All developed assays were qualified with accuracy and precision tested over the method range.

**Quantification of Cefepime and Taniborbactam in HFIM Media**

**Syneos Health: Cartridge Compatibility Methods**

Cefepime and taniborbactam in CAMHB were quantified over a range of 100–100,000 ng/mL using a high-performance liquid chromatography tandem mass spectrometry (LC-MS/MS) method. The internal standards were deuterated d_4_-taniborbactam for taniborbactam and deuterated d_8_-cefepime for cefepime. A Raptor Biphenyl (50 × 2.1 mm, 5 µm) analytical column was used to achieve separation between the two analytes. Mobile phase (MP) A and MP B were 10 mM ammonium formate in water (pH 3.5) and 50/50 (v/v) acetonitrile/methanol, respectively.

The gradient started with 5% MP B then gradually increased to 50% MP B in 2 minutes and was then held at 95% MP B between 2.01 and 3 minutes before returning to 5% MP B after 3.01 minutes until the end of the gradient (4.5 minutes). The flow rate was 0.5 mL/min throughout the gradient. Samples were processed by a protein precipitation method and the analytes and internal standards were detected in positive mode using electrospray ionization source with multiple reaction monitoring. The precursor to product ion transitions for analytes used were mass-to-charge ratio (m/z) 390.2 to 372.3 and 481.2 to 324.2 for taniborbactam and cefepime, respectively; the transitions for internal standards were m/z 394.2 to 376.3 and 489.2 to 396.2 for d_4_-taniborbactam and d_8_-cefepime, respectively.

**Venatorx: Confirmatory HFIM Pharmacokinetic Sample Methods**

Taniborbactam in CAMHB was quantified over a range of 50 to 50,000 ng/mL using an ultraperformance LC-MS/MS method. The internal standard was deuterated d_4_-taniborbactam. A Waters Acquity HSS T3 (50 × 2.1mm, 1.8 µm) was used for this method. MP A and MP B were 0.1% formic acid in water and 0.1% formic acid in acetonitrile, respectively. The gradient started with 5% MP B and stayed at 5% MP B for 0.3 minute then gradually increased to 95% MP B between 0.3 and 1 minute and was then held at 95% MP B for 0.5 minute before returning to 5% MP B after 1.51 minutes until the end of the gradient (2 minutes). The flow rate was 0.6 mL/min throughout the gradient. Samples were processed by a protein precipitation method, and the analyte and internal standard were detected in positive ion mode using electrospray ionization source with multiple reaction monitoring. The precursor to product ion transitions used were m/z 390.3 to 191.1 and 394.3 to 191.1 for taniborbactam and d_4_- taniborbactam, respectively.

**Quantification of Meropenem and Vaborbactam in HFIM Media**

**Syneos Health: Cartridge Compatibility Methods**

Meropenem and vaborbactam in 50/50 (v/v) CAMHB/1M 3-(N-morpholino) propanesulfonic acid (MOPS buffer) were quantified over a range of 100–100,000 ng/mL using a high-performance LC-MS/MS method. The internal standards were deuterated d_6_-meropenem for meropenem and cefepime for vaborbactam. A Raptor Biphenyl analytical column was used to achieve the separation between the two analytes. MP A and MP B were 10 mM ammonium formate in water (pH 3.5) and 50/50 (v/v) acetonitrile/methanol, respectively. The gradient started with 10% MP B then gradually increased to 40% MP B in 2 minutes and was then held at 95% MP B between 2.01 and 3 minutes before returning to 10% MP B after 3.01 minutes until the end of the gradient (4.5 minutes). The flow rate was 0.5 mL/min throughout the gradient. The samples were processed by a protein precipitation method and the analytes and internal standards were detected in positive mode using electrospray ionization source with multiple reaction monitoring. The precursor to product ion transitions for analytes used were m/z 384.4 to 141.0 and 280.2 to 220.1 for meropenem and vaborbactam, respectively; the transitions for internal standards were m/z 390.4 to 147.0 and 481.2 to 324.2 for meropenem and vaborbactam, respectively. The quality control acceptance criterion was ±15% at each level except at 100 ng/mL (±20%).

**Venatorx: Confirmatory HFIM Pharmacokinetic Sample Methods**

Vaborbactam in 50/50 (v/v) CAMHB/1 M MOPS was quantified over a range of 50–50,000 ng/mL using an ultra-performance LC-MS/MS method. The internal standard was rizatriptan and the column was a Waters Acquity C18 (2.1 × 50 mm, 1.7 µm). Mobile phase (MP) A and MP B were 5 mM ammonium acetate (pH 4) and acetonitrile, respectively. The gradient started with 5% MP B until 0.2 minute then increased to 95% MP B in 1.8 minutes and was then held at 95% MP B between 2.0 and 2.8 minutes before returning to 5% MP B after 2.90 minutes until the end of the gradient (3.4 minutes). The flow rate was 0.5 mL/min throughout the gradient. The samples were processed by a protein precipitation method through an Ostro Protein Precipitation & Phospholipid Removal plate. Analyte and internal standard were detected in positive mode using electrospray ionization source with multiple reaction monitoring. The precursor to product ion transitions for analytes used were m/z 298.3 to 280.1 and the transition for the internal standard was m/z 270.2 to 201.2. The developed assay was qualified with accuracy and precision test over the method range with a lower limit of quantification of 50 ng/mL. The quality control acceptance criterion was ±20% at each level.

**Quantification of Ceftazidime and Avibactam in HFIM Media**

**Syneos Health: Cartridge Compatibility Methods**

Ceftazidime and avibactam in CAMHB were quantified over a range of 100–100,000 ng/mL using a high-performance LC-MS/MS method. The internal standards were ^13^C_5_-avibactam for avibactam and d_6_-ceftazidime for ceftazidime, respectively. A Waters Atlantis dC18 analytical column was used to achieve the separation between the two analytes. Mobile phase (MP) A and MP B were 0.1% formic acid in water and 50/50 (v/v) acetonitrile/methanol, respectively. The gradient started with 0% MP B then gradually increased to 30% MP B in 3.5 minutes and was then held at 90% MP B between 3.51 and 4.5 minutes before returning to 0% MP B after 4.51 minutes until the end of the gradient (6 minutes). The flowrate was 0.5 mL/min throughout the gradient. The samples were processed by protein precipitation method and the analytes and internal standards were detected in positive mode using electrospray ionization source with multiple reaction monitoring. The precursor to product ion transitions for analytes used were m/z 264.1 to 96 and 547.2 to 468.2 for avibactam and ceftazidime, respectively; the transitions for internal standards were m/z 269.1 to 96 and 553.2 to 474.2 for avibactam and ceftazidime, respectively.

**Venatorx: Confirmatory HFIM Pharmacokinetic Sample Methods**

Avibactam in CAMHB was quantified over a range of 100 to 50,000 ng/mL using a high-performance LC-MS/MS method. The internal standard was d_8_-cefepime. A Waters Acquity HSS T3 (50 × 2.1mm, 1.8 µm) was used for this method. Mobile phase (MP) A and MP B were 0.1% formic acid in water and 0.1% formic acid in acetonitrile, respectively. The gradient started with 0.5% MP B and stayed at 5% MP B for 0.4 minute then gradually increased to 95% MP B between 0.4 and 0.8 minute and was then held at 95% MP B until 1.5 minutes before returning to 0.55% MP B after 1.51 minutes until the end of the gradient (2 minutes). The flow rate was 0.6 mL/min throughout the gradient. Samples were processed by a protein precipitation method and the analyte and internal standard were detected in negative ion mode and positive ion mode, respectively, using electrospray ionization source with multiple reaction monitoring. The precursor to product ion transitions used were m/z 264.1 to 96 and 489.1 to 396 for avibactam and d_8_-cefepime, respectively.

**Supplementary Appendix C: Qualification experiment with an MBL-positive strain, *P. aeruginosa* AR 0054 (VIM-4-positive)**

HFIM qualification was performed with an MBL-positive strain, *P. aeruginosa* AR 0054 (VIM-4-positive), treated with ceftazidime-avibactam in CAMHB supplemented with or without 20 µg/mL EDTA (i.e., zinc-chelator) to examine whether the activity of the MBL impacts the efficacy of ceftazidime-avibactam. The ceftazidime-avibactam MIC against AR 0054 was 2 µg/mL with EDTA and 128 μg/mL without EDTA. Ceftazidime-avibactam was selected as it was the most similar β-lactam/β-lactamase inhibitor to cefepime-taniborbactam with respect to the β-lactam component (i.e., a cephalosporin) and β-lactamase inhibitor spectrum (i.e., ESBL coverage) that was also commercially available at the time of the study. Two starting inocula of approximately 10^8^ and 10^7^ CFU total were evaluated. The highest inoculum density at which ceftazidime-avibactam with EDTA prevented the outgrowth of less susceptible subpopulations to ceftazidime-avibactam with EDTA was set as the target inoculum for the planned evaluation of simulated human cefepime-taniborbactam exposures against this strain (i.e., similar in concept to a “non-inferiority” clinical trial design).

When EDTA was added to the HFIM arms and treated with the simulated human ceftazidime-avibactam dose, the inoculum was held to a density of 6.2 and 6.4 log_10_ CFU/mL, respectively, at hour 6, for both the 10^8^ CFU inoculum (**Figure S1(A)**) and 10^7^ CFU inoculum (**Figure S1 (B)**) treatment arms. However, greater reductions were observed from 24- to 96-hour time points in the 10^7^ CFU inoculum arm (**Figure S1**). Moreover, a trend toward regrowth was observed from hour 72 to hour 96 in the 10^8^ CFU inoculum arm (**Figure S1 (A)**), and the entire population (6.0 log_10_ CFU/mL) demonstrated elevated ceftazidime-avibactam (with EDTA) MIC as evidenced by equal density of colonies on the non-selective and 4× MIC supplemented CAMHA. In contrast, no colonies were recovered on 4× MIC supplemented plates from the 10^7^ CFU inoculum arm (**Figure S1 (B)**), while the total bacterial burden of 3.3 log_10_ CFU/mL at hour 96 was recovered on nonselective plates.

**Figure S1: Hollow fiber infection model qualification experiments with ceftazidime-avibactam ± EDTA against *P. aeruginosa* AR 0054 (VIM-4-positive) strain**

1. 10^8^ CFU inoculum

1. 10^7^ CFU inoculum

*Figure S1 legend:*

HFIM experiments evaluating the activity of ceftazidime-avibactam alone (black, open box) and ceftazidime-avibactam plus EDTA (black, filled box) are listed. Ceftazidime-avibactam without EDTA was evaluated against the 10^7^ inoculum only and served as the growth control for both experiments. Subpopulations that grew on 4× MIC CAMHA (with EDTA) following treatment with ceftazidime-avibactam plus EDTA (blue star) are depicted. The dashed line represents the lower limit of detection.

**Abbreviations**: AR, denotes a strain sourced from the Centers for Disease Control and Prevention and Food and Drug Administration Antimicrobial Resistance (AR) Isolate Bank; ATCC, American Type Culture Collection; CFU, colony forming unit; CZA, ceftazidime-avibactam; EDTA, ethylenediaminetetraacetic acid; HFIM, hollow fiber infection model; MIC, minimum inhibitory concentration

**Figure S2: Concentration-time profiles of targeted and measured concentrations of cefepime**

***Figure S2 legend***

Cefepime target concentrations were verified in preliminary cartridge compatibility studies using non-infected media. These studies confirmed that the targeted cefepime human concentration-time profile (gray line) was achieved in the central reservoir (open circles) and equilibrated with the extracapillary space (black squares). The dashed line at 8 µg/mL represents the maximum cefepime-taniborbactam MIC determined among the HFIM study strains (range: 0.25–8 µg/mL).

**Abbreviations**: HFIM, hollow fiber infection model

**Figure S3: Concentration-time profiles and measured concentrations of avibactam and vaborbactam within HFIM assessments.**

1. **Avibactam**


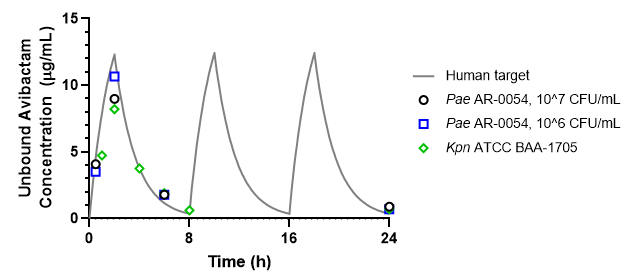


1. **Vaborbactam**


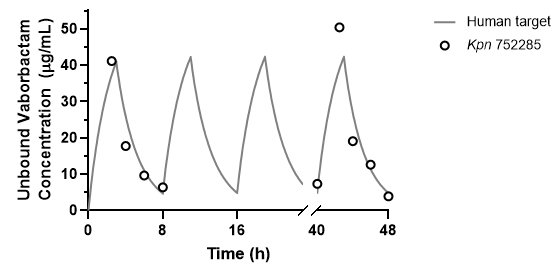


**Abbreviations**: ATCC, American Type Culture Collection; CFU, colony forming unit; *Kpn*, *Klebsiella pneumoniae*; *Pae*, *Pseudomonas aeruginosa*
